# Topological Analysis of Electron Density in Graphene/Benzene and Graphene/hBN

**DOI:** 10.3390/ma18081790

**Published:** 2025-04-14

**Authors:** Igor Fedorov

**Affiliations:** Kemerovo State University, Krasnaya 6, 650000 Kemerovo, Russia; ifedorov@kemsu.ru

**Keywords:** graphene, hBN, first principles calculation, van der Waals interaction, electron densities, quantum theory of atoms in molecules

## Abstract

Graphene is a modern material with unique properties which is used to create prototypes of gas, mechanical, and biological sensors. The non-covalent functionalization of graphene expands the scope of its practical application. Therefore, graphene-based van der Waals heterostructures are used to create various electronic devices. Thus, for a better understanding of physicochemical properties of graphene-based materials, it is necessary to study the role of van der Waals interactions in such structures in greater detail. This paper presents a study of the electron properties of structures such as graphene/benzene, graphene/graphene, and graphene/hBN within the framework of density functional theory with van der Waals interactions. Topological properties of electron densities were studied using the quantum theory of atoms in molecules. Visualization of the regions of van der Waals interaction and calculation of the charges of the regions describing the van der Waals interaction were possible due to the use of the reduced density gradient function. A comparison of the characteristics of the critical points of the electron density of graphene/graphene and graphene/hBN van der Waals heterostructures was also performed, which allowed us to compare the parameters of van der Waals interactions between different configurations of the systems under study.

## 1. Introduction

In 2004, Novoselov and Geim’s group proposed a method for fabricating graphene [1]. Currently, doping graphene with molecules makes it possible to obtain new systems with unique properties for practical applications [2,3,4]. Prototypes of various graphene-based sensors have been created [5,6,7,8], including those for medical use [9]. Studies of the system properties where van der Waals (vdW) interactions play a key role are of great interest [10]. At present, van der Waals heterostructures [11,12] and molecular crystals [13,14,15] have been studied in sufficient detail.

The properties of van der Waals heterostructures have been actively studied over the last decade. It turned out that it is possible to create new materials with unique properties by combining different 2D structures [16,17,18]. A new direction called “twistronics” was created by rotating layers relative to each other [19]. It should be noted that vdW heterostructures can be multilayered, which results in a large number of different variants of structures with different properties. The development of methods for the synthesis of new 2D materials [20,21] also opens possibilities for creating new vdW heterostructures.

Currently, the synthesis of graphene on an industrial scale is impossible, which restricts its practical application. However, graphene synthesis from aromatic molecules on substrates, such as Si or Cu, is one potential vector of development [22,23], as in this case, a structure, which can have wider practical application, is synthesized. As an additional advantage, the above mentioned substrates have been long used in the industry to manufacture electronic devices.

Pristine graphene, being extremely hydrophobic, has certain restrictions such as weak electrochemical activity, easy agglomeration, and difficult processing. Due to the non-covalent modification of graphene, these problems can be eliminated and, consequently, the areas of its practical application expand [24,25]. The physical adsorption of molecules on the graphene surface, caused by vdW interactions, preserves graphene’s atomic and electronic structure. Atomistic modeling of the non-covalent modification of graphene, on the other hand, contributes to a better understanding of physicochemical properties of such systems, as well as ways to improve them.

The mechanisms of bond formation between the components of the system are largely understudied. Thus, it is worthwhile to study electron density between graphene and benzene molecules. The benzene molecule has one carbon ring, so it is the simplest aromatic molecule, but its properties are transferable to larger aromatic molecules. Two benzene molecules represent a system where van der Waals interactions can be studied, while with the help of the obtained results, the properties of more complex systems containing carbon rings can be explained. For this reason, benzene dimers have been repeatedly studied using the high-accuracy CCSD(T) method [26,27]. The obtained results are included in the S22 database [28], which is used for testing modern accounting schemes of van der Waals interactions within the framework of DFT.

Currently, density functional theory (DFT) is a powerful tool to study various properties of different systems. Local density approximation (LDA) and global gradient approximation (GGA) have made it possible to investigate crystal properties, leading to the development of computational methods to study solids. Many different density functionals have already been developed, while new variants also continue to be developed [29,30,31,32]. The van der Waals forces originate from nonlocal interactions between electrons; this is the reason why they were not taken into account in the early density functional approximations (LDA and GGA). The vdW interactions were not taken into account in the B3LYP and PBE0 hybrid functionals either. The MP2 and CCSD(T) high-accuracy quantum chemistry methods take into account van der Waals interactions, so it is possible to make accurate computation of the properties of benzene and naphthalene dimers [33]. However, these methods require quite high computational costs (~N^6^–N^7^). Due to the unique properties of molecular crystals and van der Waals heterostructures, as well as the growth of computational capabilities, various schemes for taking into account van der Waals interactions within the framework of density functional theory have been developed. Various computational schemes have now been developed to account for vdW interactions. The DFT-D [34], vdW-DF [35,36,37,38,39,40] rVV10 [41], MBD [42], and Tkatchenko-Scheffler [43] schemes are currently widely used. These schemes continue to be developed and improved.

There is now a general understanding of bond formation between planar organic molecules consisting of benzene rings. The study of mechanisms of chemical bond formation is a traditional area of quantum chemistry, while solids are much less studied. There are a large number of organic molecules containing a benzene ring, so the graphene/benzene system represents a prototype of systems consisting of graphene and aromatic molecules. The purpose of this study is to investigate the mechanism of bond formation between an aromatic molecule and graphene. At the same time, this system is in a stable configuration due to vdW interactions. Thus, the study of the distribution and redistribution of electron density between graphene and benzene molecules is important for a better understanding of the role of van der Waals interactions in such systems.

The adsorption of organic and non-organic molecules on carbon materials has been studied within DFT for quite a long time [44,45,46,47,48,49,50]. These are early research works in which vdW interactions were often not taken into account, as these schemes had not been developed yet. Thus, benzene adsorption on carbon nanotubes within the framework of LDA was studied by Tournus et al. [51]. DFT-LDA does not take into account van der Waals interactions, but reproduces the geometric parameters of the system under study, where they play a key role [51]. Hernández et al. studied the graphene–phenol interactions within DFT-PBE framework [52]. Berland et al. used van der Waals density functionals to study the properties of benzene on graphene [53]. In this study, the equilibrium distance between graphene and benzene is predicted to be 3.25–3.6 Å. De Moraes et al. [54] calculated within DFT-LDA that the binding energy and equilibrium distance are equal to −2.97 kJ/mol and 3.75 Å, respectively. Thus, it makes sense to compute the equilibrium configuration of graphene/benzene using modern accounting schemes of intermolecular interactions.

Currently, van der Waals heterostructures are being widely studied [55,56]. To better understand the mechanisms of atomic friction, the distribution of electron density between the layers is studied [57,58]. Repulsion and van der Waals attraction forces act between the layers. Thus, the displacement of the layers relative to each other leads to a change in the interaction between them, which is manifested in a change in the binding energy and equilibrium distance. The van der Waals heterostructure of hBN has been studied many times [56,59,60,61]. Four configurations have been studied: I–IV. Configuration I had a hexagonal configuration (AA), where the carbon atoms are located above the boron atoms or nitrogen atoms. The II and III had a Bernal configuration (AB), where the C atoms of one graphene sublattice are located above the N atoms (configuration II) or B atoms (configuration III), while the C atoms of the other graphene sublattice are located above the center of the BN hexagon. Configuration IV can be obtained by translating the h-BN layer in configuration I along the C–C bond direction by a distance of 1/6 of the graphene lattice constant.

The Quantum Theory of Atoms in Molecules (QTAIM) is used to describe various properties of molecules and crystals [62,63,64,65]. QTAIM allows one to determine the quantitative characteristics of chemical bonds between atoms, which makes it easier to compare them to each other. Zhikol et al. studied the stacking interaction for the benzene dimer in the QTAIM framework [63]. The authors showed that correlation equations based on topological properties of the electron densities allow the π···π-stacking interaction energy between benzene rings to be calculated. QTAIM provides data for a deeper understanding of the mechanisms of chemical bond formation, and is used in solid state chemistry to study various phenomena. At the same time, this approach has also been repeatedly used to analyze electron densities obtained by experimental methods [66,67,68,69,70]. Gajda et al. achieved the first successful determination of quantitative charge distribution in crystal under high pressure [71].

However, within the framework of this theory, weak vdW interactions are not described as informatively as covalent interactions. For this reason, the development of methods that would effectively describe the van der Waals interactions within the framework of the theory continues [72,73]. It is of interest to use methods developed within the framework of the QTAIM to study the interaction of an aromatic molecule and graphene. Moreover, topological analysis of electron density can be used to study van der Waals heterostructures, which will contribute to a better understanding of the mechanisms of interaction between the layers.

## 2. Computational Details

A plane-wave pseudopotential approach within DFT was used to compute the total energy. The Quantum ESPRESSO V.7.2 (QE) [74] with the functional of Perdew, Burke, and Ernzerhof (PBE) [75] was used to carry out the computations. The ultrasoft pseudopotentials of the Rabe-Rape-Kaxiras-Joannopoulos type were used for calculations [76]. The structures were optimized with the Broyden–Fletcher–Goldfarb–Shanno (BFGS) method [77]. The vacuum gap used to eliminate interactions between adjacent layers was 25 Å. To study the properties of benzene/graphene, the supercell consisted of 6 × 6 × 1 graphene primitive unit cells. The energy cutoff equaled 55 Ry. The Monkhorst–Pack scheme was used for the Brillouin zone sampling [78]. The **k**-point grids were 3 × 3 × 1 and 20 × 20 × 1 for benzene/graphene and hBN/graphene, respectively.

Geometry relaxation was completed when all components of all forces were smaller than 0.1 mRy (a.u.)^−1^. I used DFT-D3(BJ) [34,79,80,81]. In this scheme, the empirical potential was added to the exchange–correlation potential, and the total energy is given by

(1) $$E_{\mathrm{DFT}‐D}=E_{\mathrm{KS}‐\mathrm{DFT}}+E_{\mathrm{disp}},$$

where *E*_KS-DFT_ is the Kohn–Sham energy and *E*_disp_ is a dispersion correction [34].

The dispersion energy is

(2) $$E_{\mathrm{disp}}=-\frac{1}{2}\sum_{A\neq B}s_{6}\frac{C_{6}^{\mathrm{AB}}}{R_{\mathrm{AB}}^{6}+{\left[f(R_{\mathrm{AB}}^{0})\right]}^{6}}+s_{8}\frac{C_{8}^{\mathrm{AB}}}{R_{\mathrm{AB}}^{8}+{\left[f(R_{\mathrm{AB}}^{0})\right]}^{8}}$$

with

(3) $$f(R_{\mathrm{AB}}^{0})=a_{1}R_{\mathrm{AB}}^{0}+a_{2},$$

(4) $$R_{\mathrm{AB}}^{0}=\sqrt{\frac{C_{8}^{\mathrm{AB}}}{C_{6}^{\mathrm{AB}}}}.$$

Here, the sum is over of all atom pairs in the crystal. The *C*_6_ and *C*_8_ are isotropic dispersion coefficients for atom pair AB, and *R*_AB_—internuclear distance. The *s*_6_ and *s*_8_ are global (functional dependent) scaling factors. Detailed information and values of all parameters can be found in the original works [34,79]. DFT-D3(BJ), as well as other schemes which take into account the vdW interactions, contribute to the correct prediction of the structural and elastic properties of molecular crystals, which is confirmed by the agreement between theoretical and experimental data [14,82,83].

The binding energies of the benzene molecule on graphene were calculated using the following formula:

*E* = *E*_benzene/graphene_ − *E*_pristine graphene_ − *E*_benzene,_ (5)

where *E*_benzene/graphene_, *E*_pristine graphene_, and *E*_benzene_ are the total energies of the fully relaxed systems containing the benzene molecule on the graphene, only graphene, and only benzene molecule, respectively.

The electronic structures of the systems were calculated using the CRYSTAL17 [84,85], using the PBE0 hybrid functional [86] and DZVP basis set [87]. The **k**-point grids are 8 × 8 × 1 and 20 × 20 × 1 for benzene/graphene and hBN/graphene, respectively. In this case, a linear combination of atomic orbitals (LCAO) is used as basis sets. Since the number of basis functions is determined by the number of atoms and does not depend on the volume of the unit cell, it makes it easier to study the electronic structure of systems consisting of surface and adsorbed molecules. Topological analysis of the electron density was performed using the TOPOND14 [88], according to the Quantum Theory of Atoms in Molecules (QTAIM) developed by Bader et al. [62]. Topological analysis of electron density can be performed using electron density values calculated on a uniform grid over the space, while the $\rho (\vec{r})$ intermediate values are interpolated. TOPOND is included into the CRYSTAL17, which makes it possible to calculate the necessary values during the topological analysis process. Visualization and analysis of the properties of critical points (CPs) was performed using the CritPlot [89]. Visualization for Electronic and Structural Analysis software (VESTA, series 3) [90] was used for the visualization of the three-dimensional electron density distribution.

## 3. Results and Discussion

### 3.1. Benzene Molecule on a Graphene

**Figure 1:**
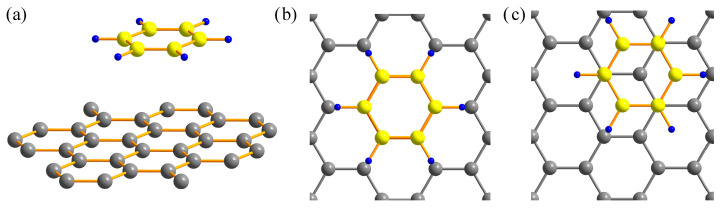
The adsorption benzene molecule on a graphene sheet (**a**). Benzene on graphene on hollow site (**b**). Benzene on graphene on top site (**c**). Carbon atoms are shown in gray, when belonging to the graphene, and yellow when belonging to the benzene. Hydrogen atoms are shown in blue.

Figure 1 shows the adsorption of a benzene molecule on graphene on hollow (H-benzene/graphene) and top (T-benzene/graphene) sites. Equilibrium distances are given in Table 1. The different schemes of the dispersion-corrected DFT (DFT-D3(BJ), rVV10, vdw-df2-b86r) predict the distances that have reasonable agreement with each other. The equilibrium distance is 3.48 Å for hollow, and 3.37 Å for top site benzene on graphene. The binding energy of T-benzene/graphene (−0.42 eV) is smaller than that of H-benzene/graphene (−0.39 eV), hence this configuration is more stable. The experimental value of the cohesive energy for the adsorption of benzene on the graphene surface is −0.50 ± 0.08 eV [91]. Similar arrangement of carbon rings relative to each other in other aromatic compounds also leads to large values of binding energy. Calculations performed using CCSD(T) reveal that such an arrangement of carbon rings relative to each other corresponds to the most stable configuration of benzene dimer [27]. This is referred to as “graphite-like”. A similar arrangement of carbon rings is observed in graphite crystal and molecular crystals of hydrocarbons. This is due to the fact that the *p*_z_-orbitals of the carbon rings overlap less, which results in reduced Pauli repulsion and allows molecules to come closer together.

Note that pure DFT-LDA is sometimes used nowadays, which predicts correct values. Thus, in a recent work, the authors used LDA with the Perdew and Zunger (PZ) parametrization [54]. I also performed the calculation within the DFT-PZ and found that the binding energy and the equilibrium distance are equal to 3.22 Å and −0.31 eV, respectively. The obtained data are consistent with the literature data [50,54]. At the same time, the calculation within the pure DFT-PBE predicts the binding energy equal to −0.04 eV. The equilibrium distance is 4.12 Å. Thus, pure DFT-LDA can be used to study the interactions between graphene and a molecule. Although the binding energy differs from the experimental value slightly more than in the case of PBE-D3, it has quite a reasonable value.

**Figure 2:**
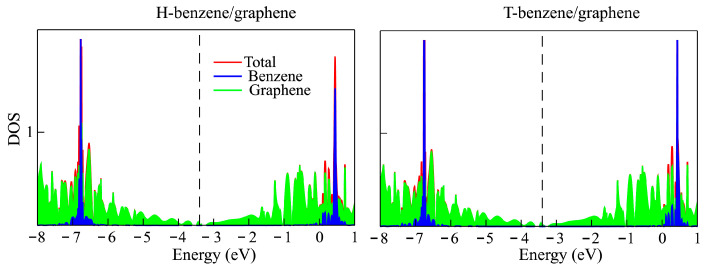
Total and projected DOS for H-benzene/graphene and T-benzene/graphene. The top of the valence band is indicated by the Fermi level (dashed line).

The calculated densities of states (DOSs) are demonstrated in Figure 2. The PDOS shows that the graphene electrons are the main contributors to the formation of the top valence and lower unoccupied bands. The band gap of graphene is zero. The band gap of H-benzene/graphene and T-benzene/graphene is 0.002 and 0.012 eV, respectively.

**Figure 3:**
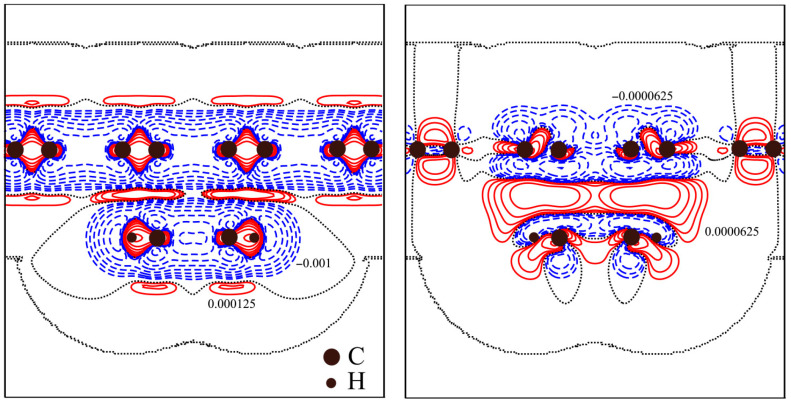
Deformation (**left**) and difference (**right**) electron density map of H-graphene/benzene system (a.u., in logarithmic scale). The positive values are marked by a solid line, and negative by a dashed line, while the null contour is marked by a dotted line.

Figure 3 shows deformation and difference electron density distribution for H-graphene/benzene. The deformation density is the difference between electron density $\rho (\vec{r})$ for graphene/benzene and the sum of the atomic electron densities. The deformation density shows how the electron density of isolated atoms is deformed when a chemical bond is formed between atoms. It has been established that when a chemical bond in the molecule is formed, the charge leakage occurs in the C–C, C-H bond line. The distribution of deformation density is typical for *sp*^2^ hybridization. The non-hybridized *p_z_*–orbitals are perpendicular to the plane of carbon rings and are responsible for the so-called π–stacking. The study of the distribution of deformation density has permitted us to establish that a charge leakage from carbon atom π–regions occurs, situated perpendicular to the plane of the carbon ring.

The difference electron density $\Delta \rho (\vec{r})$ is the difference between electron density for $\rho (\vec{r})$ for graphene/benzene and the sum of the electron densities of pristine graphene and isolated benzene molecules:

$$\Delta \rho \left(\vec{r}\right)=\rho_{\frac{graphene}{benzene}}\left(\vec{r}\right)-\rho_{graphene}\left(\vec{r}\right)-\rho_{benzene}\left(\vec{r}\right)$$

The difference electron density shows the changes in electron density due to the interaction of graphene and benzene. The $\Delta \rho (\vec{r})$ distribution demonstrates that charge delocalizes from the *p*_z_-orbitals of carbon atoms and accumulates in the space between benzene molecule and graphene. Electron density increases between atoms to compensate for their repulsion. The redistribution of electron density is insignificant because van der Waals interactions are much weaker than covalent bonds.

The Hirshfeld charges [92,93] of the benzene molecule atoms are calculated. The charges of the carbon and hydrogen atoms are −0.053 and 0.049 |*e*|, respectively. In the T-benzene/graphene case, the charges of three carbon atoms are −0.054 |*e*|, and the other three atoms have a charge of −0.052 |*e*|. The charges of the hydrogen atoms are 0.049 |*e*|. Therefore, the charge of the benzene molecule in both cases is −0.024 |*e*|. Thus, the charge transfer between the graphene and benzene molecules is practically non-existent (−0.001 |*e*|/atom).

The analysis of electron density distribution allows us to establish the mechanisms of chemical bond formation. For a better understanding and comparison of quantitative characteristics, it is convenient to use QTAIM. The electron density is a scalar quantity; by calculating $\nabla \rho \left(\vec{r}\right)$, we can perform its topological analysis. Critical points (CPs) are also determined as a result of this analysis. In total, there are four types of critical points used for quantitative comparison of chemical bonding parameters. There are three types of critical points in the graphene/benzene system. The ring critical point (RCP) is located in the center of each carbon ring. The values of $\rho \left(\vec{r}\right)$ and $\Delta \rho \left(\vec{r}\right)$ at the corresponding CPs are practically the same for both forms of graphene/benzene. The $\rho \left(\vec{r}\right)$ and $\Delta \rho \left(\vec{r}\right)$ values in the center of the benzene molecule are 0.023 and 0.178 a.u., respectively. These values are the same for H- and T-graphene/benzene and isolated benzene molecule. These values are the highest for the graphene/benzene system among all RCPs.

**Figure 4:**
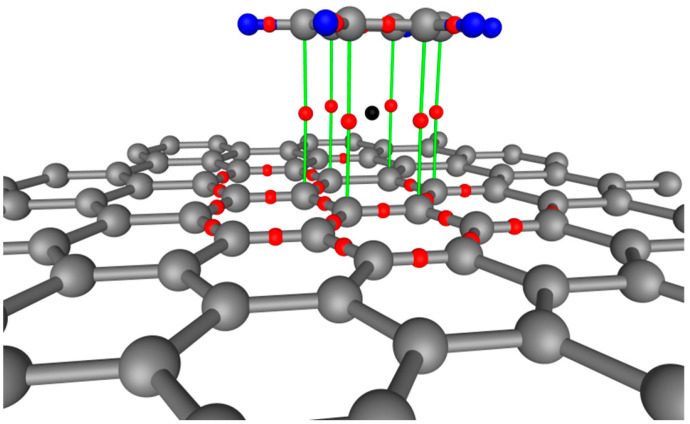
Some critical points (red and black spheres) of the H-graphene/benzene.

Due to its high symmetry, H-graphene/benzene is easier to analyze. There are almost six equivalent bond critical points (BCPs). In this case, CPs describing the interaction between graphene and benzene are located on the shortest lines connecting carbon atoms from benzene and graphene (Figure 4). The length of these lines is ~3.48 Å each and correspond to the distance between the benzene molecule and graphene. The values of $\rho \left(\vec{r}\right)$ and $\Delta \rho \left(\vec{r}\right)$ in these CPs are 0.0032 and 0.0148 a.u., respectively. For example, these values at the CP located on the C-C bond in graphene are 0.2897 and −0.6798 a.u., respectively. Thus, the values differ from each other by several orders of magnitude. Since $\Delta \rho \left(\vec{r}\right)>0$, it means that the electron density between benzene and graphene delocalizes. The eigenvalues of the Hessian for H-graphene/benzene have the following values: λ_1_ = −0.00171, λ_2_ = −0.00124, and λ_3_ = 0.01775. At the critical point, the value of electron density is very small, |λ_1_|/λ_3_ = 0.096 < 1, $\Delta \rho \left(\vec{r}\right)>1$, which corresponds to the interaction of systems with closed shells. This result is related to the Pauli principle, since graphene and benzene molecule both have filled shells, they weakly overlap in the interaction region. The value of λ_2_ < 0 has a small value at the CP. Thus, the characteristics of the CP correspond to the van der Waals interaction between the graphene and benzene molecules. The critical points located between the carbon atoms of the benzene molecule and graphene for T-graphene/benzene have the following parameters: $\rho \left(\vec{r}\right)=0.0041\;a.u.$, $\Delta \rho \left(\vec{r}\right)=0.0179\;a.u.$, λ_1_ = −0.00218, λ_2_ = −0.00185, and λ_3_ = 0.02195. Thus, the mechanism of bond formation between graphene and benzene is similar in both cases. In the case of the T-graphene/benzene, the displacement of benzene molecules leads to a decrease in the overlap of *p*_z_-orbitals of carbon atoms, which reduces the repulsion of benzene molecules and grapheme; as a result, the van der Waals forces bring them closer by shortening the distance between them.

The reduced density gradient, *s*, is used to visualize van der Waals interactions [72], and has the following form:

$$s=\frac{1}{{2\left(3\pi^{2}\right)}^{\frac{1}{3}}}\frac{\left|\nabla \rho \right|}{\rho^{\frac{4}{3}}}$$

**Figure 5:**
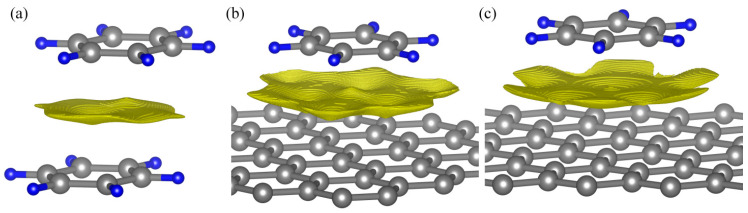
The intermolecular interaction surface in the benzene dimer (**a**), benzene on graphene on top (**b**) and hollow (**c**) site. The isosurface was generated for $\rho (\vec{r})<0.005\;a.u.$, r_s_ = 1.3, 1.3, and 1.5 a.u. for the benzene dimer and the benzene on graphene on the top and hollow site, respectively.

Due to this function, it is possible to determine weak interaction regions. Figure 5 shows the *s*(ρ) plot for graphite-like benzene dimers and benzene molecules on graphene. The *s*(ρ) function allows us to visualize small electron density values that correspond to vdW interactions and exclude the values associated with covalent bonds. It can be clearly seen that the regions around the critical points under consideration are responsible for weak interaction between graphene and benzene molecules.

It is possible to determine the surface corresponding to the van der Waals interaction by specifying the values of the electron density and the gradient function of reduced density (Table 2). The van der Waals interaction is very weak; therefore, the corresponding values of the electron density are also small. Figure 4 shows the surface *S*, which corresponds to the values $\rho (\vec{r})<0.005\;a.u.$ and *r*_s_ = 1.3 a.u. for the graphite-like (Gr-like) benzene dimer. The *S* is an orientable closed surface with a Euler characteristic of 2. For example, a sphere has the same characteristics. The Euler characteristic for a torus is 0. The volume *V*_S_ bounded by the surface *S* is 2.42 Å^3^. The electronic charge *Q*_S_ is 0.0268 |*e*|. Figure 4 shows the surface *S*, which corresponds to the values $\rho (\vec{r})<0.005\;a.u.$ and rs = 1.1 for the T-benzene/graphene. The volume *V*_S_ = 6.45 Å^3^, *Q*_S_ = 0.1089 |*e*|. For example, if $\rho (\vec{r})<0.005\;a.u.$ and *r*_s_ = 1.3, then *V*_S_ = 4.75 Å^3^, *Q*_S_ = 0.0811 |*e*|. The corresponding values for the H-benzene/graphene are as follows: $\rho (\vec{r})<0.005\;a.u.$, *r*_s_ = 1.5 a.u., *V*_S_ = 6.28 Å^3^, *Q*_S_ = 0.0950 |*e*|. Thus, the average value of the electron density ($\rho (\vec{r})=Q/V$) inside *S* is approximately equal to 0.02 a.u. This value of the electron density is several orders of magnitude smaller than the values of the electron density in a covalent bond.

Integral characteristics make it possible to determine the quantitative characteristics of the van der Waals interaction and compare the properties of different systems. The charge *Q*_S_ between benzene and graphene is greater than that between two benzene molecules. Thus, the interaction between graphene and a benzene molecule is greater than between two benzene molecules. This is due to the fact that each atom of a benzene molecule in a dimer forms twelve pairs with atoms of another molecule. In the case of a graphene/benzene system, the number of pairs is greater. Formally, the number of pairs is equal to infinity, but with increasing distance, the interaction will tend to zero. The charge increases when transitioning from one configuration to another, which is accompanied by a decrease in the equilibrium distance, as well as a decrease in the binding energy of the system by 0.03 eV.

### 3.2. Graphene/Graphene Van Der Waals Heterostructure

Two graphene sheets can form an AA and AB structure. In the case of vdW heterostructures, both structures are infinite. In AA form, the carbon atoms of one layer are located above carbon atoms of the other layer. In AB form, graphene layers are arranged as in graphite crystal. The binding energy and equilibrium distance are 0.072 eV and 3.65 Å for AA form. As for AB form, *E*_bind_ = 0.0879 eV and *d* = 3.40 Å. The critical point describing the interaction between graphene sheets is located in the middle of the line connecting carbon atoms. The values of $\rho (\vec{r})$ and $\Delta \rho (\vec{r})$ are 0.0020869 (0.0039621) and 0.010608 (0.017358) a.u. for AA (AB) form, respectively. The computed lattice parameters of crystalline graphite are as follows: a = 2.461 and c = 6.748 Å. The values of $\rho (\vec{r})$ and $\Delta \rho (\vec{r})$ at the critical point are 0.0041855 and 0.018215 a.u., respectively. Thus, the critical point parameters for the AB form are slightly less than those in the graphite crystal. Since graphene contains only carbon atoms, the analysis is simpler.

The transition from AA to AB stacking can be considered the simplest model for analyzing friction mechanisms on an atomic level. The displacement of a graphene sheet from the AA to AB position is followed by a decrease in the binding energy and the convergence of the sheets. The quantitative study of the interactions between the layers can be performed using topological analysis. The values of the electron density and its Laplacian increase at the critical point. This being said, the Laplacian of the electron density has a positive value between graphene sheets. This means that the charge density is deconcentrated between graphene sheets. Positive values of the Laplacian of the electron density allow us to identify areas between layers where van der Waals interactions play a key role. The electron density near atoms is tightly bound to the nuclei of atoms and changes slightly when the layers are shifted relative to each other. Meanwhile, the electron density located inside the corrugated surfaces changes with the displacement of the layers.

**Figure 6:**
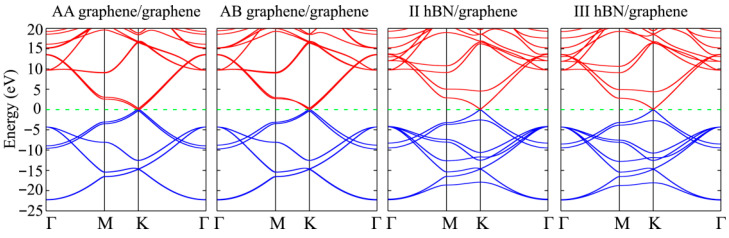
Band structures of graphene/graphene and hBN/graphene within PBE0 approximation. The dashed line denotes the top of the valence band (Fermi level).

Figure 6 presents the computed band structures for AA and AB graphene/graphene. The top of the valence band is the reference point. The calculated band gaps of AA graphene/graphene and AB graphene/graphene are 0.001 and 0 eV, respectively.

### 3.3. Graphene/hBN Van Der Waals Heterostructure

**Figure 7:**
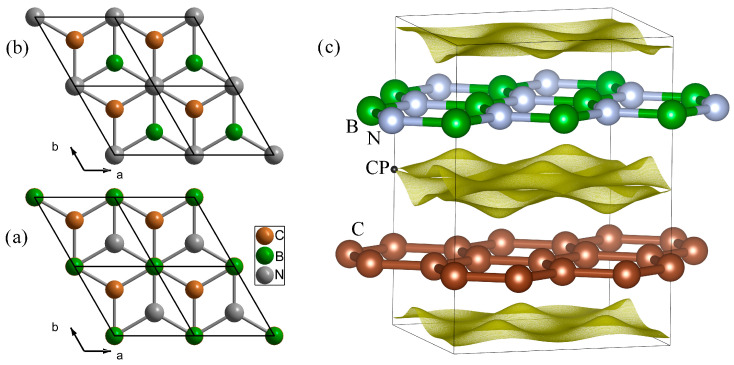
(**a**) Configuration II of the hBN. (**b**) Configuration III of the hBN. (**c**) Isosurface of the electron density ($\rho \left(\vec{r}\right)=\rho (CP)$). For convenience, only one nonequivalent point (CP) is shown.

Now, let us study two forms of graphene/hBN vdW heterostructure (Figure 7). In this case, there are three types of atoms (boron, carbon, and nitrogen) in the structure. The equilibrium distance and the corresponding binding energy for configuration II are 3.35 Å and 0.0885 eV, respectively. As for configuration III, *E*_bind_ = 0.0749 eV and *d* = 3.50 Å. These values are in good agreement with the literature data [56,59,60,61]. Note that using pure LDA also predicts correct values [59].

The charge transfers between graphene and hBN are 0.003 and 0.001 |*e*|/atom for configuration II and III, respectively. Thus, the van der Waals interaction causes an insignificant charge transfer between the layers. Figure 6 shows the band structures for two hBN/graphene configurations. The electronic structure of hBN/graphene heterostructure was studied by experimental methods [94,95], including by NanoARPES [96]. The theoretical results are consistent with the experimental data. The calculated band gap of II hBN/graphene and III hBN/graphene equals 0.057 and 0.030 eV. The energy gap of configuration II computed with linearized augmented plane waves plus local orbitals (LAPW + lo) is equal to 0.056 meV [97].

The critical point describing the interaction between graphene and hBN is located on the line connecting carbon and boron (carbon and nitrogen) atoms for configuration II (III). The distance between the CP and carbon atom is 1.70 and 1.73 Å for configuration II and III, respectively. Thus, the critical point is closer to graphene for configuration II. As for configuration III, the critical point lies closer to the hBN plane. The values of $\rho (\vec{r})$ and $\Delta \rho (\vec{r})$ are 0.0038399 (0.0032639) and 0.018025 (0.014870) for configuration II (III), respectively. The value of $\Delta \rho (\vec{r})$ characterizes the bond strength. Thus, the interaction between the layers of configuration II is stronger than that of configuration III. At the same time, the value of the Laplacian of the electron density is greater than the value of two graphene layers. This can be explained by the fact that the boron atom is smaller than the carbon atom. Therefore, since the repulsion between the atoms will be less, the arrangement of the layers in configuration II allows them to approach each other and shorten the distance. This leads to an increase in the van der Waals interaction and the binding energy between the layers.

Figure 5 shows the isosurface of the electron density where the critical point is located. This is where the two surfaces that belong to the graphene and hBN layers come into contact. This is due to the fact that, by definition, the requirement $\nabla \rho \left(\vec{r}\right)=0$ for the critical point is fulfilled. Therefore, the critical point parameters can be used to analyze the interactions between 2D layers, for example, to analyze atomic friction, and to explain the nature of the mechanical properties of vdW heterostructures. Thus, this approach can be considered as a simpler model that allows ($\rho \left(\vec{r}\right)\leq \rho_{CP}\left(\vec{r}\right)$) to identify the contact region between the layers of van der Waals heterostructures.

## 4. Conclusions

The equilibrium configurations for the modified graphene have been calculated using DFT-D3(BJ). The accurate account for vdW interactions made it possible to calculate the binding energies for the systems under investigation, which are in good agreement with the known experimental and theoretical data. Band structures for vdW heterostructures graphene/graphene and graphene/hBN were studied. The parameters of critical points describing vdW interactions for graphene/benzene, graphene/graphene, and graphene/hBN were calculated using QTAIM. The results of this study demonstrate that quantitative description of the interactions between components of a graphene-based system can be performed using QTAIM.

The distances between the benzene molecule and graphene planes equal 3.48 and 3.37 for H- and T-graphene/benzene, respectively. The analysis of electron density characteristics in critical points between graphene and benzene demonstrates that displacement of benzene molecule leads to the increase in electron density, resulting in distance shortening between benzene and graphene.

The use of the reduced density gradient function made it possible to determine areas where van der Waals interaction plays a key role. The obtained results contribute to a better understanding of the formation of grapheme-based and aromatic molecules-based structures. Since the results for molecular crystals of hydrocarbons are transferable, similar mechanisms should be expected from interactions between graphene and more complex hydrocarbon molecules, such as naphthalene, anthracene, etc. The NCI between graphene and a benzene molecule can be quantitatively described using a reduced density gradient function. This approach can be used for the quantitative evaluation of the interactions between a molecule and a layer, which is relevant, for example, for the creation of 2D material-based sensors.

A topological analysis of the electron density of graphene/graphene and graphene/hBN van der Waals heterostructures has been performed. The quantitative parameters of the interaction between the layers of heterostructures have been calculated. The values of the Laplacian of the electron density at the critical point correlate with the binding energy of van der Waals heterostructures, which makes it possible to study interactions between layers. Thus, the calculation of the parameters of the electron density at critical points allows us to conduct quantitative comparative analysis of the interaction between the components of van der Waals systems. Since QTAIM is based on the distribution of the electron density, but does not depend on the method of obtaining it (experimentally or theoretically), this approach can be used to predict stable configurations.

## Figures and Tables

**Table 1:** Equilibrium distances (Å) of the benzene/graphene computed within different schemes.

	DFT-D3(BJ)	rVV10	MBD	vdw-df2-b86r
H-benzene/graphene	3.48	3.45	4.18	3.45
T-benzene/graphene	3.37	3.38	4.13	3.35

**Table 2:** Calculated volumes (*V*_S_) and charges (*Q_S_*) of intermolecular interaction surfaces ($\rho (\vec{r})<0.005\;a.u.$).

	*r_s_*, a.u.	*V*_S_, Å^3^	*Q_S_*, |*e*|
Gr-like dimer benzene	1.3	2.42	0.0268
T-benzene/graphene	1.3	6.45	0.1089
H-benzene/graphene	1.5	6.28	0.0950

## Data Availability

Dataset available on request from the authors.
